# Children with Respiratory Disease Associated with Metapneumovirus in Hong Kong

**DOI:** 10.3201/eid0906.030009

**Published:** 2003-06

**Authors:** J.S. Malik Peiris, Wing-Hong Tang, Kwok-Hung Chan, Pek-Lan Khong, Yi Guan, Yu-Lung Lau, Susan S. Chiu

**Affiliations:** * The University of Hong Kong, Queen Mary Hospital, Hong Kong SAR, China

**Keywords:** Human metapneumovirus, children, respiratory infections, wheezing, research

## Abstract

Human metapneumovirus (HMPV) is a newly discovered pathogen thought to be associated with respiratory disease. We report the results of a study of 587 children hospitalized with respiratory infection over a 13-month period. HMPV was detected in the nasopharyngeal aspirates from 32 (5.5%) children by reverse transcription-polymerase chain reaction. HMPV infection was associated with clinical diagnoses of pneumonia (36%), asthma exacerbation (23%), or acute bronchiolitis (10%). When compared to those with respiratory syncytial virus infection, children with HMPV infection were older, and wheezing was more likely to represent asthma exacerbation rather than acute bronchiolitis. HMPV viral activity peaked during the spring-summer period in Hong Kong. Phylogenetically, all HMPV virus strains from Hong Kong belonged to one of the two genetic lineages previously described. HMPV contributed to 441.6 hospital admissions per 100,000 population <6 years of age.

Globally, respiratory infections in childhood are a leading cause of disease, contributing to absenteeism and economic strain through use of healthcare resources (*1*). In the developing world, respiratory infections are also a major cause of childhood death, although the contribution of viruses to such deaths is unclear (*2*). Respiratory syncytial virus (RSV) and influenza are recognized as important contributors to hospitalization (*3**–**5*). Despite sensitive diagnostic methods, an etiologic agent still cannot be identified in a portion of children with acute respiratory infection (*6*). Human metapneumovirus (HMPV) is a recently discovered respiratory virus belonging to the family *Paramyxovidiridae* (*7*,*8*), and its clinical significance is still being defined. After its initial discovery in the Netherlands, HMPV has been detected in respiratory specimens from patients of all ages in a number of countries, e.g., Canada, Australia, United Kingdom, and Finland (*9*–*14*). In children, HMPV has been reported to cause disease similar to that of RSV; signs and symptoms range from severe cough to bronchiolitis and pneumonia. However, a detailed analysis of clinical signs in children is lacking. Furthermore, HMPV has not been reported in tropical or subtropical regions. Routine immunization of children against influenza is now under active consideration by the Advisory Committee on Immunization Practices, and vaccines for RSV are being developed. Therefore, defining the role of the newly emerging HMPV is important in childhood respiratory disease.

## Materials and Methods

### Study Design

The Hong Kong Special Administrative Region is located within the tropics but has a subtropical climate. Within this region of Hong Kong Island are 288,371 persons <18 years of age and 84,018 <6 years of age (based on 2001 Census data). Two publicly funded hospitals of the Hospital Authority of Hong Kong, Queen Mary Hospital and Pamela Youde Nethersole Eastern Hospital, provide 90% of all acute pediatric hospital care (admission ratio 1:1.65). At Queen Mary Hospital, a nasopharyngeal aspirate is routinely collected for viral investigation from all children hospitalized with acute respiratory disease.

We investigated a systematic sample of children (<18 years of age) admitted with acute respiratory infection to Queen Mary Hospital during a 13-month period. From August 2001 to March 2002, all children admitted to Queen Mary Hospital with symptoms of respiratory infection on one fixed day each week were included in this study of HMPV infection. From April through August 2002, study enrollment was increased to twice weekly. The results of virologic diagnosis of HMPV were not available to the attending pediatricians. The clinical features of children identified to have HMPV infection were compared with age-matched controls with influenza A or RSV infection.

### Viral Diagnosis

Nasopharyngeal aspirates from patients were tested for RSV, influenza A and B, adenovirus, and parainfluenza types 1, 2, and 3 by culture and immunofluorescent antigen detection as previously described (*15*,*16*). An aliquot of these aspirates was snap-frozen for testing for HMPV. Viral RNA were extracted by using the RNAeasy kit (QIAGEN GmbH, Hilden, Germany) and tested by reverse transcription-polymerase chain reaction (RT-PCR) with primers to the viral L gene as previously described (*7*). All positive results were confirmed by retesting with a nested RT-PCR with primers to regions of the M gene conserved between HMPV and avian pneumovirus (*8*).

Twelve microliters of viral RNA was amplified in 20 μL volumes containing the following components: 2 mM Tris-HCL (pH7.5), 10 mM NaCL, 0.01 mM EDTA, 0.11 mM DTT, 0.001% NP-40, 0.5 mM each of the four deoxynucleotide triphosphates, 7.5 ng random primers (Invitrogen, Life Technology, Carlsbad, CA), and 20 U of Superscript II Rnase H^-^ Reverse Transcriptase (Invitrogen, Life Technology). The reactions were allowed to proceed in a thermocycler programmed to incubate for 50 min at 42°C and 3 min at 94°C cycler (Perkin-Elmer Cetus, Gouda, the Netherlands). Five microliters of cDNA was used for PCR amplification reaction with 0.5 μM of two primers (sense 5′-CATGCCCACTATAAAAGGTCAG-3′ and anti-sense 5′-CACCCCAGTCTTTCTTGAAA-3′), corresponding to the sequence of the L gene of metapneumovirus. Samples were amplified by heating at 95°C for 12 min, 30 cycles of 94°C for 1 min, 60°C for 1 min, 72°C for 1 min, and a final of extension period of 7 min at 72°C. Similarly, 5 μL of cDNA was used for a first PCR amplification reaction with 0.5 μM of two degenerated primers (sense 5′-AARGTSAATGCATCAGC-3′ and anti-sense 5′-CAKATTYTGCTTATGCTTTC-3), corresponding to the sequence of the matrix gene of metapneumovirus, which can detect both avian and human metapneumovirus. Samples were amplified in similar reaction mixture previously described by heating at 95°C for 12 min, 30 cycles of 94°C for 30 s, 60°C for 30 s, 72°C for 1 min, and a final of extension period of 7 min at 72°C. A 5-μL aliquot of the first PCR product was transferred into a second PCR tube for nested PCR by using two inner specific M gene primers for HMPV (5′-ACACCTGTTACAATACCAGC-3′ and 5′-GACTTGAGTCCCAGCTCCA-3′). The reaction mixture was subjected to a further 95°C, 12 min, 30 cycles of 94°C, 55°C, and 72°C for 1 min each, and a final of extension period of 7 min at 72°C. PCR products were analyzed by electrophoresis in a 2% (w/v) agarose gel and stained with 0.5 μg/mL of ethidium bromide. The sizes of L gene and M gene nested PCR products were 171 bp and 201 bp, respectively. Only specimens with a positive result for both tests were regarded as confirmed positive.

The 171-bp fragment of the RT-PCR–amplified product of the viral L gene was sequenced for phylogenetic analysis. The PCR-amplified DNA was sequenced by using the Big Dye Terminator Cycle Sequencing Ready Reaction kit (Applied Biosystems, Foster City, CA). Briefly, PCR products were purified by using the QIA quick PCR Purification Kit (QIAGEN GmbH) according to manufacturer’s instruction. A total of 35 to 50 ng of purified PCR products were mixed with two tubes containing 1.6 pmol (forward and reverse), 4 μL Terminator reaction mix (containing deoxy- and dideoxy-nucleotides, and modified Taq polymerase), and made up to a final volume of 10 μL with MilliQ water. Cycle sequencing was performed in the thermocyler with profile consisted of a 96°C denaturation step for 30 s, followed by an annealing temperature of 50°C for 15 s and extension temperature of 60°C for 4 min, for a total of 25 cycles. Unincorporated dye terminators and nucleotides were removed by using the DyeEx kit (QIAGEN GmbH). The procedure was performed according to the user manual of the package. The DNA template was denatured at 94°C for 4 min and stored in ice, ready for sequencing on a Perkin-Elmer 377 XL DNA sequencer (Applied Biosystems). DNA sequences were aligned by using the Clustal X software, and phylogenetic analysis was conducted by using MEGA (v. 2.1, Arizona State University, Temple, AZ). The distances for the multiple aligned DNA sequences were estimated by using the Jukes-Cantor method and a phylogenetic tree constructed by using neighbor-joining method. The tree was subjected to bootstrap test (100 replicates) and bootstrap values >40 are shown.

### Chart and Radiographic Review

All medical records were reviewed by a pediatrician (S.S.C.). At the end of the study, the chest radiographs were reviewed by a pediatric radiologist (P.L.K.), who knew that these children had a cough and febrile illness but did not know the clinical or microbiologic diagnoses.

## Results

### Epidemiology

**Figure 1:**
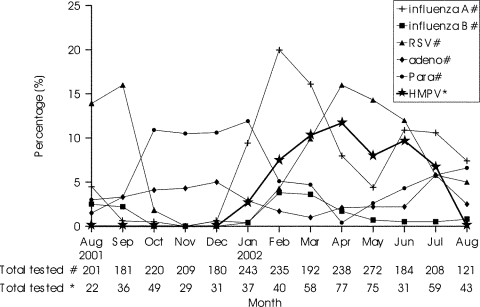
Number of patients tested and percentage positive for human metapneumovirus and the seasonality of other common respiratory viruses during the study period. Data for human metapneumovirus are based on a subset of patients admitted to Queen Mary Hospital (see Methods); data for the other respiratory viruses are based on all children admitted.

**Figure 2:**
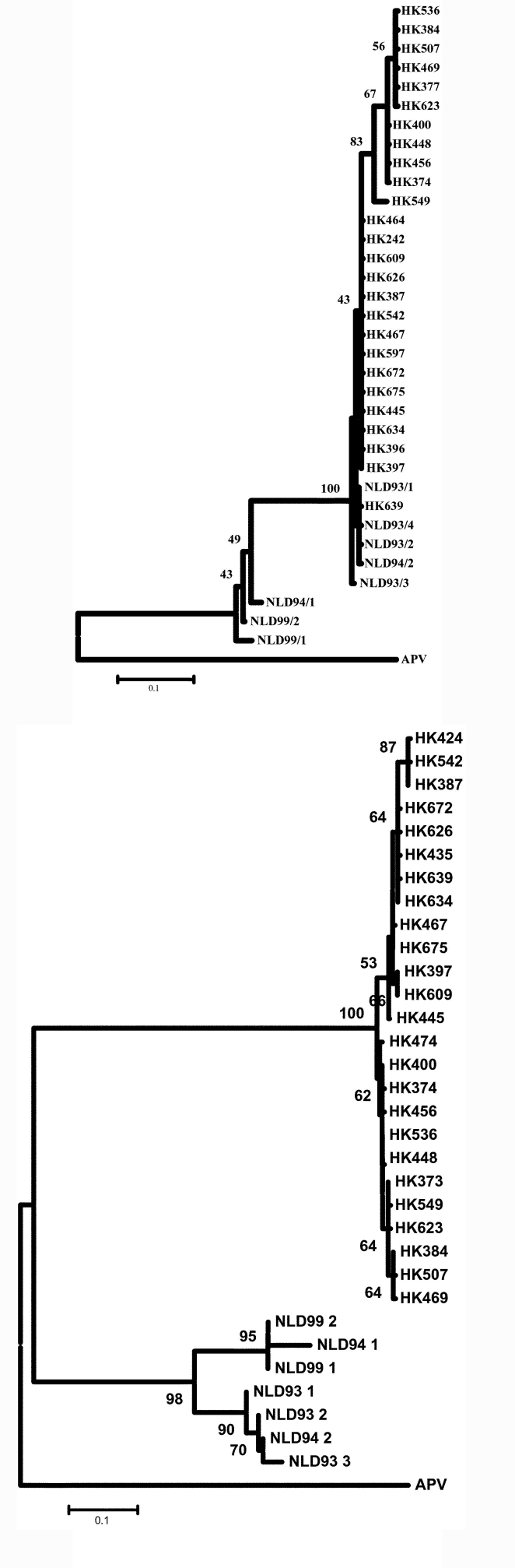
Phylogenetic tree of the human metapneumovirus a) L gene and b) F gene. Viruses detected in Hong Kong are prefixed HK, and the sequences have been deposited in GenBank under accession numbers AY294849 through to AY294870. Other viral sequences were obtained from GenBank. Abbreviations used: APV, avian pneumovirus; NL, the Netherlands.

Since obtaining an nasopharyngeal aspirates to test for common respiratory viruses is a routine diagnostic procedure, all children with acute respiratory symptoms admitted on the study days were enrolled. A total of 587 patients were studied; 302 were enrolled in the first 7 months of the study with patients being admitted on one fixed day of each week. Another 285 patients were enrolled during the last 6 months, with patients admitted on two fixed days of the week recruited to the study. The study sample represented 15.5% of all 3,787 acute pediatric admissions to Queen Mary Hospital and 22.9% of all 2,563 admissions for acute respiratory disease. Thirty-two (5.5%) children had HMPV virus RNA detected in the nasopharyngeal aspirate specimen when tested by RT-PCR. In comparison, routine immunofluorescent antigen detection and viral culture documented that 8% of children had RSV (mean age 19.50 ± 17.24 months), 8% had influenza A or B (mean age 30.23 ± 22.61 months), 5% had parainfluenza (mean age 37.75 ± 36.87 months), and 3.1% had adenovirus infections (mean age 44.11 ± 53.22 months). Children with HMPV ranged from 3 months to 72 months of age (mean 31.7 ± 18.7 months) and were older than those with RSV infection (p=0.004). The preponderance of males (M:F 3:1) with HMPV was greater than that of the study group overall (M:F 1.2:1) (p=0.03). The peak of HMPV activity occurred in spring and the early summer months in 2001–02 in Hong Kong (Figure 1). The seasonality of other common respiratory viruses during the study period based on viral diagnoses of all children admitted with respiratory infections to Queen Mary Hospital showed that RSV had a similar seasonality as HMPV (Figure 2). Two children with HMPV disease had co-infection: one with adenovirus and another with influenza A. No other children had a viral co-infection.

During the first 12 months of the study, 2,563 persons were admitted for acute respiratory infections to Queen Mary Hospital. From the monthly HMPV isolation rate in our representative sample, we calculated the total of HMPV infections admitted to Queen Mary Hospital during each of the first 12 study months. We estimated that 126 children were hospitalized with HMPV during this period. On average, the ratio of acute pediatric admissions between Queen Mary Hospital and Pamela Youde Nethersole Eastern Hospital is 1:1.65. Since these two hospitals provide 90% of all hospital admissions for Hong Kong Island, we estimated that 371 persons were admitted to the hospital with HMPV of 84,018 children <6 years of age living in Hong Kong Island, resulting in a hospitalization rate of 441.6 per 100,000 population <6 years of age. As the mean duration of hospitalization per episode was 3.17 days (Table 1), we estimated HMPV accounted for 1,176 days of hospitalization per year in children <6 years of age on Hong Kong Island.

**Table 1:** Fever and duration of hospitalization of 32 children admitted with HMPV with age-matched controls with RSV and influenza A^a^

Characteristics	HMPV	RSV	Influenza A	p value^b^
	Mean (SD)	Mean (SD)	Mean (SD)	
Age (months)	31.70 (18.40)	31.75 (18.41)	31.25 (18.57)	0.99
Duration of hospitalization (days)	3.17 (1.39)^d^	2.81 (1.18)	2.44 (0.96)	0.054
Highest temperature in hospital (°C)^d^	39.20 (0.59)	39.04 (0.54)	39.23 (0.60)	0.44
Duration of fever	4.53 (2.23)	3.57 (1.83)	4.50 (1.86)	0.12

^a^HMPV, human metapneumovirus; RSV, respiratory syncytial virus. ^b^p value performed using the analysis of variance (ANOVA) test. ^c^Two children with HMPV infection were excluded from analysis. A 5-year-old girl newly diagnosed with acute lymphoblastic leukemia had coryzal symptoms and was febrile with nasopharyngeal aspirates positive for HMPV on day 2 of admission. Her total hospitalization stay for chemotherapy and *Pseudomonas* septicemia was 42 days. A 3-month-old girl was admitted for evaluation of failure to thrive and to gain weight; fever, coryza, and diarrhea developed on day 7 of hospitalization. Her total hospitalization of 10 days was also removed from analysis. ^d^This excluded children who were not febrile in the hospital: three children with HMPV, seven with RSV, and four with influenza A.

### Clinical and Laboratory Findings

All children (100%) with HMPV had a fever, and 28 (90%) had cough with sputum (Table 1 and 2). Two children with HMPV had hoarseness without stridor. Five (16.1%) children had febrile seizures; two had three seizures each. Four (12.9%) children with HMPV had a truncal rash (on the chest only in three) that was blanchable, nonpruritic, maculopapular, and transient, lasting for a few hours to a day. Two children had diarrhea not related to antibiotics. Nine (29%) children had lymphopenia (mean 0.96 ± 0.26 x 10^9^/L), and two children had elevated transaminases (ALT, AST, and GGT for the two children were 80 MU/L, 40 MU/L, 77 MU/L, and 339 MU/L, 432 MU/L, 634 MU/L, respectively). Of children with HMPV infection, 26.3% had one or more adult family contacts who also had an acute respiratory illness (Table 1).

**Table 2:** Characteristics of 32 children admitted with HMPV compared with age-matched controls with RSV and influenza A^a^

Characteristics	HMPV	RSV	Influenza A	p value^b^		
	No. positive/ total (%)	No. positive/ total (%)	No. positive/ total (%)	Overall	HMPV vs. RSV	HMPV vs. influenza A
Influenzalike illness in family contact	10/19 (52.6)	7/29 (24.1)	19/24 (79.1)	0.0003	0.29	0.37
Influenzalike illness in adult family contact	5/19 (26.3)	4/29 (13.8)	13/24 (54.2)	0.005	0.68	0.45
Febrile seizures	5/32 (15.6)	1/32 (3.1)	3/32 (9.4)	0.229	—	—
Congested pharynx	12/32 (37.5)	11/32 (34.4)	11/32 (34.4)	0.955	—	—
Rash	4/32 (12.5)	1/32 (3.1)	4/32 (12.5)	0.331	—	—
Enlarged liver	2/32 (6.3)	0/32 (0.0)	4/32 (12.5)	0.331	—	—
Otitis media	4/32 (12.5)	0/32 (0.0)	0/32 (0.0)	0.201	—	—
Diarrhea	2/32 (6.3)	1/32 (3.1)	3/32 (9.4)	0.586	—	—
Crepitations	18/32 (56.3)	14/32 (43.8)	3/32 (9.4)	0.0003	0.50	0.0007
Wheezing	9/32 (28.1)	12/32 (37.5)	2/32 (6.3)	0.0109	0.60	0.04
Asthma exacerbation	6/32 (18.8)	2/32 (6.3)	2/32 (6.3)	0.167	—	—
Acute bronchiolitis	3/32 (9.4)	10/32 (31.3)	0/32 (0.0)	0.0009	0.13	0.37
Pneumonia	12/32 (37.5)	5/32 (15.6)	1/32 (3.1)	0.0017	0.30	0.009
Abnormal chest x-ray	17/25 (68.0)	11/18 (61.1)	1/17 (5.9)	0.0002	0.89	0.0002
Lymphopenia (<1.5 x 10^9^/L)	9/31 (29.0)	2/27 (7.4)	12/29 (41.4)	0.017	0.34	1.0
Neutropenia (ANC<1 x 10^9^/L)	2/31 (6.5)	0/27 (0.0)	4/29 (13.8)	0.125	—	—
Elevated transaminases	2/15 (13.3)	0/5 (0.0)	3/11 (27.3)	0.357	—	—

^a^HMPV, human metapneumovirus; RSV, respiratory syncytial virus. ^b^p values performed using McNemar chi-square test.

A 72-month-old child with newly diagnosed acute lymphoblastic leukemia had documented HMPV infection. She had very mild coryzal symptoms for 4 days with a temperature of 38.3°C for a day; and she recovered uneventfully. Fever, coryza, and diarrhea associated with HMPV developed in one other child hospitalized for failure to thrive 6 days after sharing a room for 24 hours with a child with fever and diarrhea, who was subsequently diagnosed to have HMPV infection. This incident suggested a nosocomial transmission with an incubation period for HMPV disease of 5 to 6 days.

The clinical characteristics of patients with HMPV were compared with those of age-matched children with RSV or influenza A (Table 2). Children infected with HMPV tended to have a longer duration of fever than those with RSV, although this finding did not attain statistical significance. In comparison to RSV and influenza, patients with HMPV tended to have a longer hospital stay. However, rapid diagnostic test results for patients with influenza and RSV might influence their quicker discharge from hospital (*15*). Both HMPV and RSV infections were more likely to be associated with wheezing than were influenza infections. However, in contrast to RSV infection, the cause of wheezing in HPMV infection was often asthma exacerbation rather than acute bronchiolitis. Asthma exacerbation accounted for 66.7% of the wheezing of HMPV-infected children but only 16.7% in RSV-infected children. HMPV was at least as important as influenza as a cause of febrile seizures.

### Radiographic Findings

Children with HMPV were more likely to have requests for a chest x-ray (Table 2). Independent of specialist radiologic assessment, the attending pediatricians diagnosed bacterial pneumonia in four, atypical pneumonia in five, and viral pneumonia in three children, respectively. All chest x-rays were subsequently reviewed by the pediatric radiologist. Perihilar peribronchial thickening, perihilar patchy consolidation, or both were found in 14 patients, suggesting a viral infection. Viral or atypical pneumonia was diagnosed in one child, and viral or bacterial pneumonia was diagnosed in two. Hyperinflation was seen in five children. No child had lobar consolidation.

### Phylogenetic Data

Previous studies have shown two distinct phylogenetic lineages of HMPV (*8*). The viruses detected in Hong Kong during 2002 belonged to one of these lineages (Figure 2). The viruses from the patients with presumed nosocomial transmission were genetically identical.

## Discussion

In a representative sample of children hospitalized with acute respiratory symptoms during a 13-month period, we found HMPV virus activity in the spring and summer months. Previous studies, all from temperate regions, have reported HMPV to be a virus with a winter-spring seasonality. In contrast to temperate regions, RSV (and sometimes influenza) in Hong Kong also has a spring-summer seasonality. Surveillance over more years is needed to establish whether the seasonality of HMPV is a recurrent pattern. Our estimate of 441.6 HMPV-associated hospitalizations per 100,000 children <6 years of age annually can be compared with recent estimates of influenza-related hospitalization in Hong Kong, which ranged from 2,882 per 100,000 in children <1 year of age to 773 per 100,000 children 2–5 years of age (*5*).

From this preliminary report, HMPV appears to be an important respiratory pathogen in children, causing a wider spectrum of disease than previously appreciated. Nine of the 32 patients with HMPV had wheezing, asthma exacerbation, or bronchiolitis as a symptom. Approximately one third of patients with HMPV infection were clinically diagnosed to have pneumonia. Not all patients had chest x-rays. However, 17 of the 25 patients who did had abnormalities in their chest x-rays. Thus, a minimum estimate for radiologic abnormalities in children hospitalized with HMPV infection was 53%. Influenza has been previously reported to be a major cause of febrile seizures (*17*). We found that HMPV may also be an important cause of febrile seizures. In fact, some of the children had multiple seizures during the same episode of HMPV infection. Association of HMPV with febrile seizures, rash, diarrhea, and transaminases has not been previously reported. HMPV was previously reported to cause severe lower respiratory disease and death in children with hematologic malignancies (*12*,*14*). We documented HMPV in a child with acute lymphoblastic leukemia, but the illness was mild and self-limited, possibly because infection occurred at the time of diagnosis, before any immunosuppressive therapy was initiated.

Over half of the patients with HMPV had an influenzalike illness reported in one or more of their family contacts (all ages), while 26% reported an adult family member with an influenzalike illness. Seroepidemiologic data in the Netherlands showed that all children are seropositive for HMPV antibody by 10 years of age (*7*). Further virologic studies of family contacts may elucidate the role of household transmission of HMPV. Recurrent infection has been documented in a few children, and the virus has also been detected in adults (*7*,*9*,*11*,*12*,*14*).

When compared to age-matched children infected with RSV or influenza, a greater proportion of children with HMPV infection had lower respiratory tract involvement leading to more chest x-rays being performed. HMPV appeared to be a stronger trigger for asthma exacerbation than RSV or influenza (Table 2). In a recent study of children hospitalized with wheezing in Finland, HMPV was detected in 10 (32%) of 31 children recruited during the period of peak HMPV activity (January–April) or in 7.5% of the 132 children overall (*13*). The chemokine profile (interleukin 8 and RANTES) in nasal secretions of children infected with HMPV was different from that reported in infections with RSV.

The role of viral respiratory tract infections in acute and chronic asthma has been a subject of much research interest. Globally, the prevalence of asthma has increased, and recent data show that the prevalence of asthma in Hong Kong children 6–7 years of age is 8% (*18*). Viruses have been demonstrated to be epidemiologically associated with asthma in at least two ways. They may initiate the development of the atopic state in infants and children. Further, they cause acute exacerbations in children with established asthma. RSV and parainfluenza, with their tendency to cause bronchiolitis in infants, have been most intensively studied (*19*). Recently rhinoviruses have also been recognized as a principal trigger of asthma exacerbation in older children and adults (*20*). Our data indicate that HMPV may also be important in this regard, and its role in the pathogenesis of wheezing in the younger child, as well as the mechanism by which it produces asthma symptoms, warrants further study. Elucidation of the roles of cytokines and chemokines in asthma attacks associated with viral infections, such as HMPV, will have implications for the rational use of anti-inflammatory agents such as corticosteroids and leukotriene receptor antagonists.
